# Supplementary material for: HIV-1 Trans Infection via TNTs Is Impeded by Targeting C5aR
Source: Biomolecules. 2022 Feb 15;12(2):313. doi: 10.3390/biom12020313 (PMC8868603; doi:10.3390/biom12020313)

Figure S1. TNTs formation in a DC/CD4⁺ T cell co-culture is enhanced at 7 dpi with HIV/-C compared to lower time points. TNT quantification on HIV/HIV-C-loaded i/mDCs in co-cultures with $CD4^+$ T cells at different time points after infection (24 hours, 48 hours, 4 days and 7 days). At 7 dpi, TNTs formation is enhanced compared to the tested lower time points. Three independent experiments were performed with two replicates each samples. Each dot represents a single measurement.

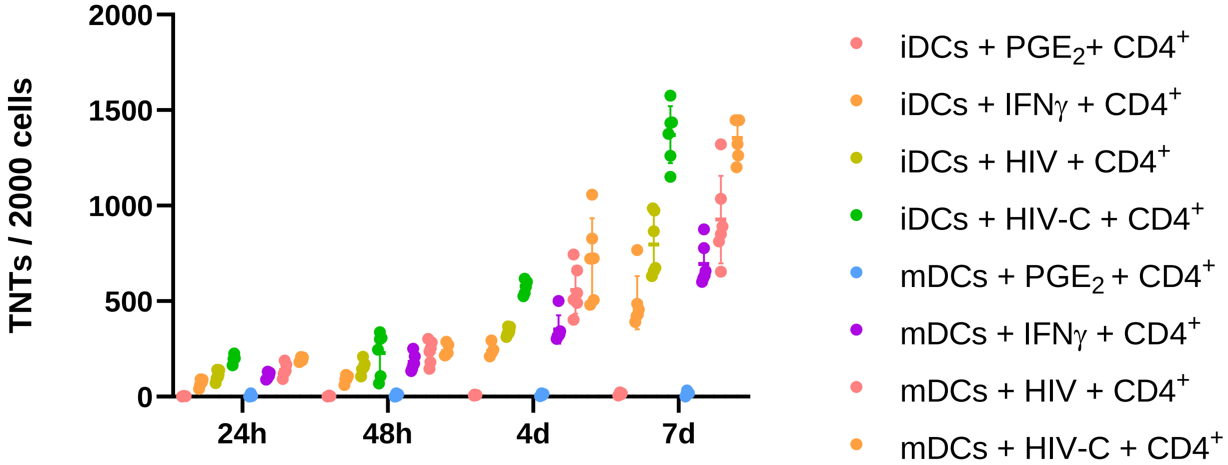

Figure S2. AraC treatment decreases $CD4^+$ T cells infection with HIV. Quantification of infection on 7 dpi supernatants from HIV/HIV-C-infected CD4+ T cells with/without 24 h treatment with 1 μM AraC. HIV-infected-CD4+ T cells show a significantly lower, moderate productive infection compared to HIV ($p < 0.0001$). HIV-infected-CD4+ T cells have a higher productive infection than HIV-C –infected-CD4+ T cells ($p < 0.0001$). Three independent experiments were performed performed with two replicates each samples. Each dot represents a single measurement. Statistical significance was analyzed using GraphPad Prism software and ordinary one-way ANOVA.

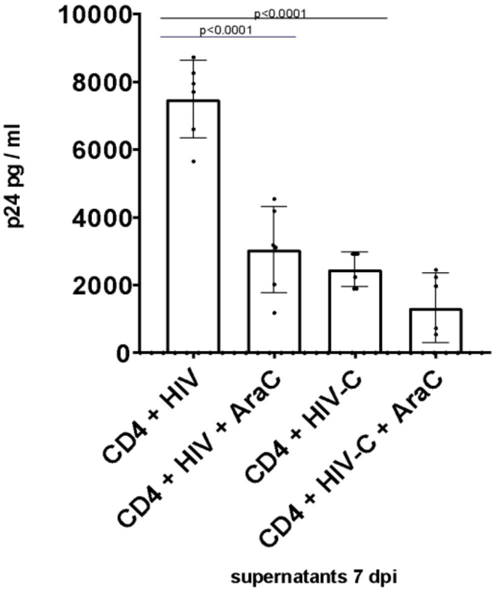

Figure S3. TNTi treatment does not show any effect on CD4+ T cells infection with HIV/-C. Quantification of infection on 7 dpi supernatants from HIV/HIV-C-infected CD4+ T cells with/without 24 h treatment with 20 µM TNTi. HIV-C-infected CD4+ T cells treated with TNTi do not show any difference in infection levels compared to HIV. Three independent experiments were performed performed with two replicates each samples. Each dot represents a single measurement. Statistical significance was analyzed using GraphPad Prism software and ordinary one-way ANOVA.

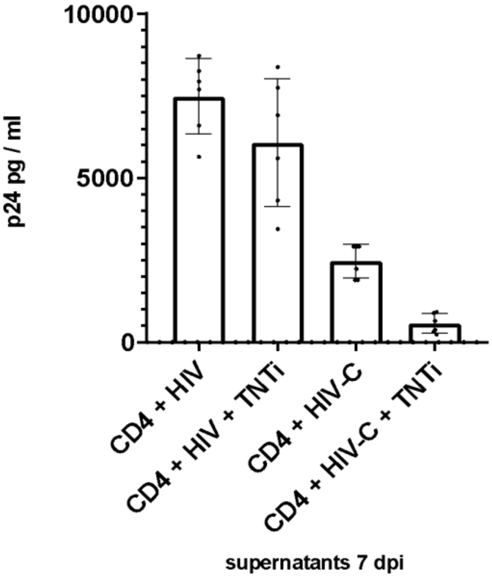

Supplement: Supplementary file 1 [file biomolecules-12-00313-s001.zip › biomolecules-1573106-supplementary.pdf]
